# USP6NL mediated by LINC00689/miR-142-3p promotes the development of triple-negative breast cancer

**DOI:** 10.1186/s12885-020-07394-z

**Published:** 2020-10-14

**Authors:** Teng Ma, Huaidong Liu, Yan Liu, Tingting Liu, Hui Wang, Fulu Qiao, Lu Song, Lin Zhang

**Affiliations:** 1 Department of Breast Surgery, Taian City Central Hospital, Taian, 271000 Shandong China; 2 Department of Oncology, Huai’an Second People’s Hospital, the Affiliated Huai’an Hospital of Xuzhou Medical University, No.62 South Huaihai Road, Huai’an, 223002 Shandong, Jiangsu China; 3 Department of Vascular Surgery, Taian City Central Hospital, Taian, 271000 Shandong China

**Keywords:** USP6NL, LINC00689, miR-142-3p, Triple-negative breast cancer

## Abstract

**Background:**

Triple-negative breast cancer (TNBC), in part because of the high metastasis rate, is one of the most prevalent causes of malignancy-related mortality globally. Ubiquitin specific peptidase 6 N-terminal like (USP6NL) has been unmasked to be implicated in some human cancers. However, the precise biological function of USP6NL in TNBC has not been defined.

**Methods:**

RNA expression was examined by real-time quantitative PCR (RT-qPCR), while USP6NL protein level was tested through western blot. Besides, cell proliferation was assessed by using colony formation assay, whereas cell apoptosis estimated by flow cytometry analysis, JC-1 assay and TUNEL assay. Transwell assays were adopted to detect the migration and invasion of indicated TNBC cells. Immunofluorescence (IF) assay evaluated epithelial-mesenchymal transitions (EMT) progress in TNBC. Further, RNA immunoprecipitation (RIP), RNA pull down and luciferase reporter assays were implemented for measuring the mutual interplay among USP6NL, miR-142-3p and long intergenic non-protein coding RNA 689 (LINC00689).

**Results:**

Elevated USP6NL level was uncovered in TNBC cells. RNA interference-mediated knockdown of USP6NL inhibited TNBC cell growth, motility and EMT. Further, USP6NL was proved as the target of a tumor-inhibitor miR-142-3p, and LINC00689 augmented USP6NL expression by absorbing miR-142-3p. Importantly, miR-142-3p deficiency or USP6NL overexpression fully abolished the inhibitory effect of LINC00689 silence on TNBC cellular behaviors.

**Conclusion:**

All data revealed the important role of USP6NL/LINC00689/miR-142-3p signaling in TNBC. The findings might provide a new and promising therapeutic biomarker for treating patients with TNBC.

## Background

Triple-negative breast cancer (TNBC) is a kind of female malignancy with high mortality [1]. Its occurrence is owing to lacking estrogen receptor and progesterone receptor, as well as elevated human epidermal growth factor receptor 2 level [2]. Even thought, immunotherapy [3, 4], radiotherapy [5] and others act as presently main therapeutic strategies for patients with TNBC, there are still great challenges for treating TNBC due to poor prognosis and heterogeneous character [6]. In past few decades, molecules like messenger RNAs (mRNAs) of protein-coding genes have been applied as promising biomarkers for TNBC treatment [7–9]. In the current study, we planned to figure out the role of USP6NL in TNBC.

The level of protein-coding genes is considered to have close association with the development of multiple tumors and diseases [10–12]. For example, strong expression levels of COL1A1 and COL1A2 was suggested to have close relation to low overall survival rate of patients with gastric cancer [13]. Up-regulation of TCF21 depressed cell proliferation and EMT in breast cancer [14]. As for the subject ubiquitin specific peptidase 6 N-terminal like (USP6NL) mentioned in this study, this gene encoded a type of GTPase-activating protein which has been reported in past few years to participate in several cancers. For instance, sun el at. suggested that USP6NL was overexpressed in colorectal cancer (CRC) cells to facilitate CRC progression [15]; USP6NL up-regulation caused glycolysis addiction in breast cancer [16]. However, the role and molecular mechanism of USP6NL in TNBC have not yet been reported or explored.

In present study, we intended to explore the function and upstream mechanism of USP6NL in TNBC.

## Methods

### Cell culture

Human TNBC cells lines (AU565, MDA-MB-453, MDA-MB-436, MDA-MB-468 and MDA-MB-231) and human normal mammary epithelial cell line (MCF-10A) were all procured from the ATCC company (Rockville, Maryland). All these TNBC cell lines are derived from metastatic tumors (metastatic site: pleural effusion or pericardial effusion). The DMEM medium (Invitrogen, Carlsbad, CA) supplementing with 10% FBS was employed for cultivating MCF-10A, AU565 and MDA-MB-453 cells. Besides, L-15 medium with 10% FBS was adopted to culture MDA-MB-436, MDA-MB-468 and MDA-MB-231 cells. All these cells were grown in 5% CO_2_ at 37 °C.

### Real-time quantitative PCR (RT-qPCR)

Total RNA was separated through adopting Trizol reagent (Invitrogen), followed by the synthesis of the first cDNA template in accordance with the protocol of suppliers (Takara Bio Inc., Shiga, Japan). Following, quantitative PCR was implemented with the SYBR R Premix Ex TaqTM II (Takara), with the result analyzed via 2^-ΔΔCt^ method. ACTB or U6 served as the control. Three bio-repeats were conducted for this experiment.

### Plasmid transfection

The shRNAs targeting LINC00689 or USP6NL, as well as the corresponding negative controls, were devised and composed by Genepharma Company (Shanghai, China). In addition, pcDNA3.1 vectors (Invitrogen) covering full-length cDNA sequence of LINC00689 or USP6NL were adopted to enhance LINC00689 or USP6NL expression, with empty vector as the negative control. The miR-142-3p mimics and NC mimics, as well as miR-142-3p inhibitor and NC inhibitor, were obtained from RiboBio (Guangzhou, China), with the final transfection concentration at 50 nM. Above plasmids were appropriately transfected into MDA-MB-468 and MDA-MB-231 cells for 48 h by utilizing Lipofectamine 2000 (Invitrogen).

### Western blot

After cells lysis via RIPA lysis buffer (Beyotime, Shanghai, China) containing protease inhibitor, total protein was acquired and the concentration was then examined with a Bio-Rad DC Protein Assay Kit (Yuwei Biotechnology, Guangzhou, China). Subsequently, protein separation was achieved by SDS-PAGE, followed by protein-transferring to PVDF membranes. Thereafter, the membranes were subjected to sealing with 5% BSA and overnight incubation at 4 °C with primary antibodies against USP6NL and β-actin. After being captured by secondary antibodies, the proteins were visualized by using chemiluminescence detection system.

### Colony formation assay

MDA-MB-468 and MDA-MB-231 cells were gathered and then planted in 96-well plate (500 cells/well). After fortnight, cells were subjected to fixation through 4% paraformaldehyde and staining via 0.1% crystal violet. In the end, the number of colonies was counted manually. The experiment was repeated no less than three times.

### TUNEL assay

After fixation, MDA-MB-468 and MDA-MB-231 cells were rinse through precooled PBS. Afterward, cells were subjected to permeation as least 15 min. In accordance with the protocol pf suppliers, TUNEL assay Kit (Beyotime, Shanghai, China) was adopted to measure apoptotic cells. Then DAPI was utilized to stain the nuclei. In the end, the apoptotic cells were captured through fluorescence microscopy (Olympus). This assay was implemented with three repeats.

### Flow cytometry analysis

Firstly, the transfected MDA-MB-468 and MDA-MB-231 cells were gathered and rinsed via precooled PBS. Then Annexin V-FITC/PI Apoptosis kit bought from BD Biosciences (San Jose, CA) was utilized to estimate the apoptosis rate of 2 × 10^5^ cells. In detail, cells were separately treated with FITC or PI for 15 min, followed by analysis through flow cytometry (BD Biosciences). The experiment was repeated no fewer than three times.

### JC-1 assay

The transfected MDA-MB-468 and MDA-MB-231 cells were subjected to cultivation in 96-well black microplate. After a night, cells were centrifuged and then the culture medium was removed. Following, cells were processed with JC-1 staining for half an hour. In the end, fluorescence microscope (Olympus) was adopted to observe and analyze the stained cells. This experiment was carried out for at least three times.

### Transwell assay

With regard to migration assay, cells in serum-free medium were put in the upper transwell chambers without Matrigel. As for invasion assay, such cells were added into chambers pre-laid with Matrigel. After that, the culture medium with 20% FBS was added in bottom chamber. After cultivating for 24 h, the residual cells in upper chamber were eliminated, while cells in the lower chamber were separately fixed and stained with methanol and crystal violet. In the end, a microscopy (Olympus) was utilized to compute the number of migrated and invaded cells. Three bio-repeats were carried out for this assay.

### Immunofluorescence (IF)

A 6-well plate was utilized to cultivate TNBC cells at the consistence of 2.5 × 10^4^ cells per well. One day later, cells were fixed at room temperature (RT) and then permeabilized by 0.1% Triton X-100. Following blockaded with 5% BSA, cells were treated with the primary antibodies against E-cadherin and N-cadherin at 4 °C. After a night, secondary antibodies (Abcam) were supplemented and incubated cells at RT for half an hour. Finally, DAPI staining was performed and cells were observed under fluorescence microscopy (Olympus). The experiment was repeated no fewer than three times.

### Subcellular fraction

MDA-MB-468 and MDA-MB-231 cells (1 × 10^6^) rinsed by precooled PBS were gathered for incubation with cell fractionation buffer and cell disruption buffer based on the PARIS™ Kit (Invitrogen). After centrifugation, LINC00689 content in the cytoplasmic or nuclear fraction was evaluated through RT-qPCR. The cytoplasmic control was ACTB and the nuclear control was U6 served. This experiment was repeated no less than three times.

### FISH assay

The devised specific RNA-FISH probe for LINC00689 was acquired from Ribobio. Based on the protocol of suppliers, cells were air-dried and cultivated with LINC00689-probe in hybridization buffer. After Hoechst staining, fluorescence microscopy was utilized to observe cells. This assay was performed with at least three repeats.

### RNA immunoprecipitation (RIP)

Firstly, cell lysates obtained via RIP lysis buffer were cultivated with human Ago2 antibody (Millipore) and magnetic beads in RIP buffer. The normal IgG antibody (Millipore) was thought as the negative control. Finally, RT-qPCR was adopted for analyzing RNA in the whole precipitates. The experiment was conducted for no fewer than three times.

### RNA pull down assay

In accordance with the protocol of suppliers, Pierce Magnetic RNA-Protein Pull-Down Kit (Thermo Fisher Scientific, Waltham, MA) was taken for determining the interaction between molecules. The prepared cell lysates were blended with Bio-USP6NL-WT/Mut, Bio-LINC00689-WT/Mut or Bio-NC, followed by being mixed with streptavidin-linked magnetic beads. In the end, RT-qPCR was utilized to analyze RNAs in the pulled down complexes. The experiment had no less than three bio-repeats.

### Luciferase reporter assay

The LINC00689 or USP6NL 3’UTR fragments which included wild-type or mutated miR-142-3p binding sites were separately inserted to the pmirGLO luciferase vector (Promega, Madison, WI). Following, above reporters were subjected to co-transfection into indicated TNBC cells with miR-142-3p mimics or NC mimics. Two days later, Luciferase Reporter Assay System (Promega) was utilized for analysis of the luciferase activity. This assay was repeated for no fewer than three times.

### Statistical analysis

All the experiments were bio-repeated no less than three times. Data were represented by means of mean ± SD. Moreover, PRISM 6 (GraphPad, San Diego, CA) software was employed for data analysis with one-way ANOVA or Student’s t-test. The *p*-value below 0.05 was considered to be statistically significant.

## Results

### USP6NL exerts pro-oncogenic functions in TNBC

**Fig. 1:**
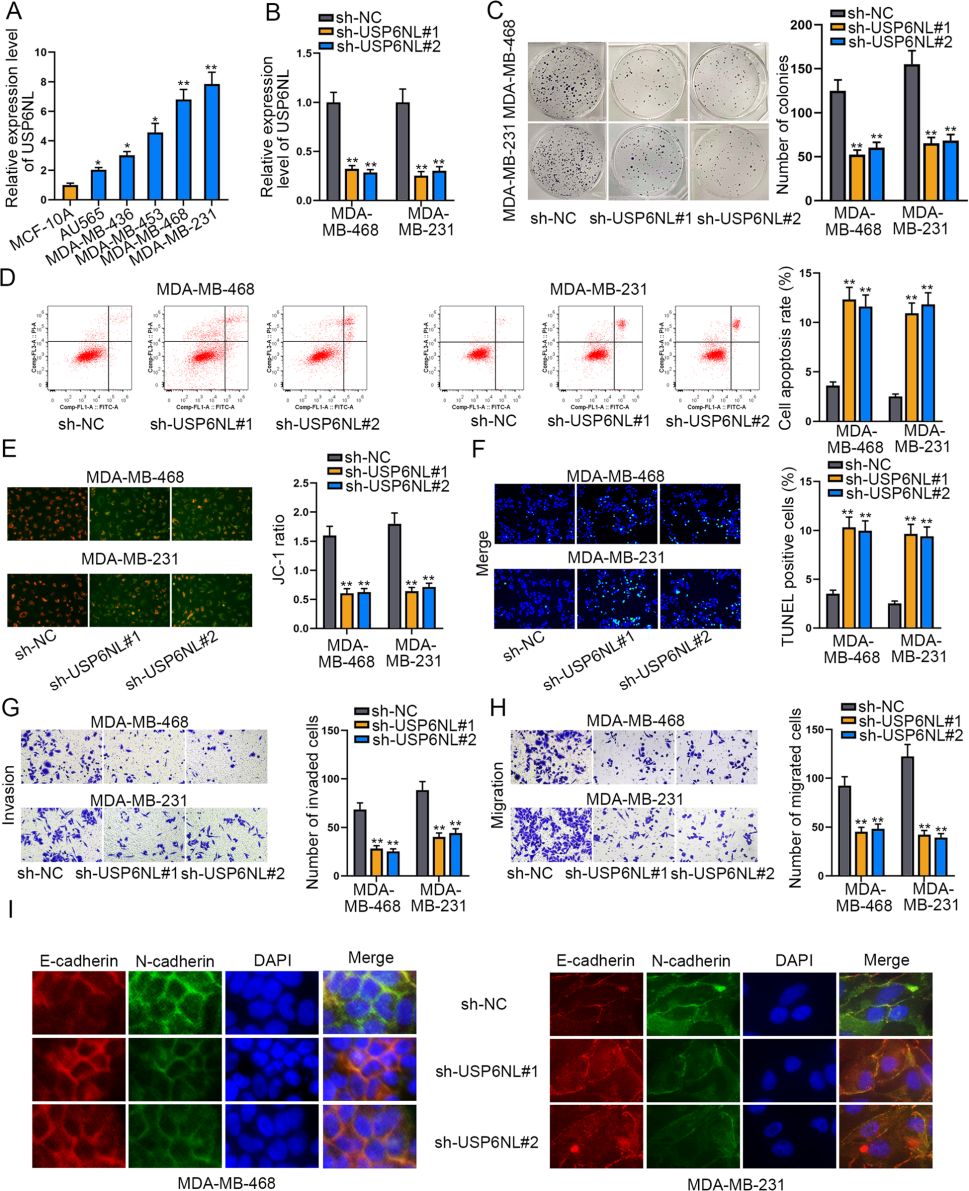
USP6NL exerts pro-oncogenic functions in TNBC by affecting cell proliferation, apoptosis, migration, invasion and EMT. **a**. The expression of USP6NL in TNBC cell lines and human normal breast cell line MCF-10A were respectively assessed with RT-qPCR. **b**. The RT-qPCR analyzed the interference efficiency of USP6NL in both cells transfected with sh-USP6NL#1/2. **c**. Colony formation assay was conducted to measure the change of cell proliferation in cells with or without USP6NL inhibition. **d**. Flow cytometry analysis determined the apoptosis rate of USP6NL-silenced cells. The X axis of the FACS dot plots meant cells labelled with FITC and the Y axis indicated cells dyed by PI. **e-f**. JC-1 assay and TUNEL assay were adopted to examine cell apoptosis under USP6NL interference or not. **g-h**. Transwell assay was used to assess the migration and invasion of TNBC cells with silenced USP6NL or not. **i**. EMT process in TNBC cells was also examined by IF analysis of E-cadherin and N-cadherin staining. *P < 0.05, **P < 0.01

To explore the role of USP6NL in TNBC, the level of USP6NL in TNBC cell lines (AU565, MDA-MB-436, MDA-MB-453, MDA-MB-468 and MDA-MB-231) and the human normal breast cell line MCF-10A were measured via RT-qPCR. The outcome displayed that USP6NL presented high expression in TNBC cell lines in comparison to MCF-10A cells (Fig. 1a). Next, we performed loss-of-function assays in MDA-MB-468 and MDA-MB-231 cells considering the highest USP6NL expression level in these two cells. Before executing functional assays, USP6NL was proved to be significantly silenced in both TNBC cells by transfecting with sh-USP6NL#1/#2, by RT-qPCR and western blot analyses (Fig. 1b **and Supplementary Figure** **1****A)**. According to the consequence of colony formation assay, knockdown of USP6NL led to apparent reduction of colony number (Fig. 1c), indicating that USP6NL silence suppressed TNBC colony-formation ability. However, the results of flow cytometry analysis mirrored that USP6NL down-regulation markedly enhanced the cell apoptosis rate (Fig. 1d). Also, the outcomes of JC-1 assay indicated that cell apoptosis was accelerated by USP6NL depletion since the JC-1 ratio was inhibited in cells with USP6NL depletion (Fig. 1e). Besides, it was indicated through TUNEL assay that silencing USP6NL could elevate TUNEL positive cell present (Fig. 1f), further suggesting cell apoptosis was promoted by knockdown of USP6NL. Moreover, it manifested that the capacities of cells migration and invasion were obviously suppressed due to USP6NL insufficient, evidenced by the declined number of migrated and invaded cells in response to USP6NL suppression (Fig. 1g-h). Furthermore, IF assay indicated that USP6NL deficiency led to increased signals of E-cadherin declined staining of N-cadherin in both TNBC cells (Fig. 1i), declaring that USP6NL had an accelerating impact on EMT process in TNBC. In sum, we confirmed that USP6NL exerts pro-oncogenic functions in TNBC.

### MiR-142-3p acts as the upstream of USP6NLin TNBC

**Fig. 2:**
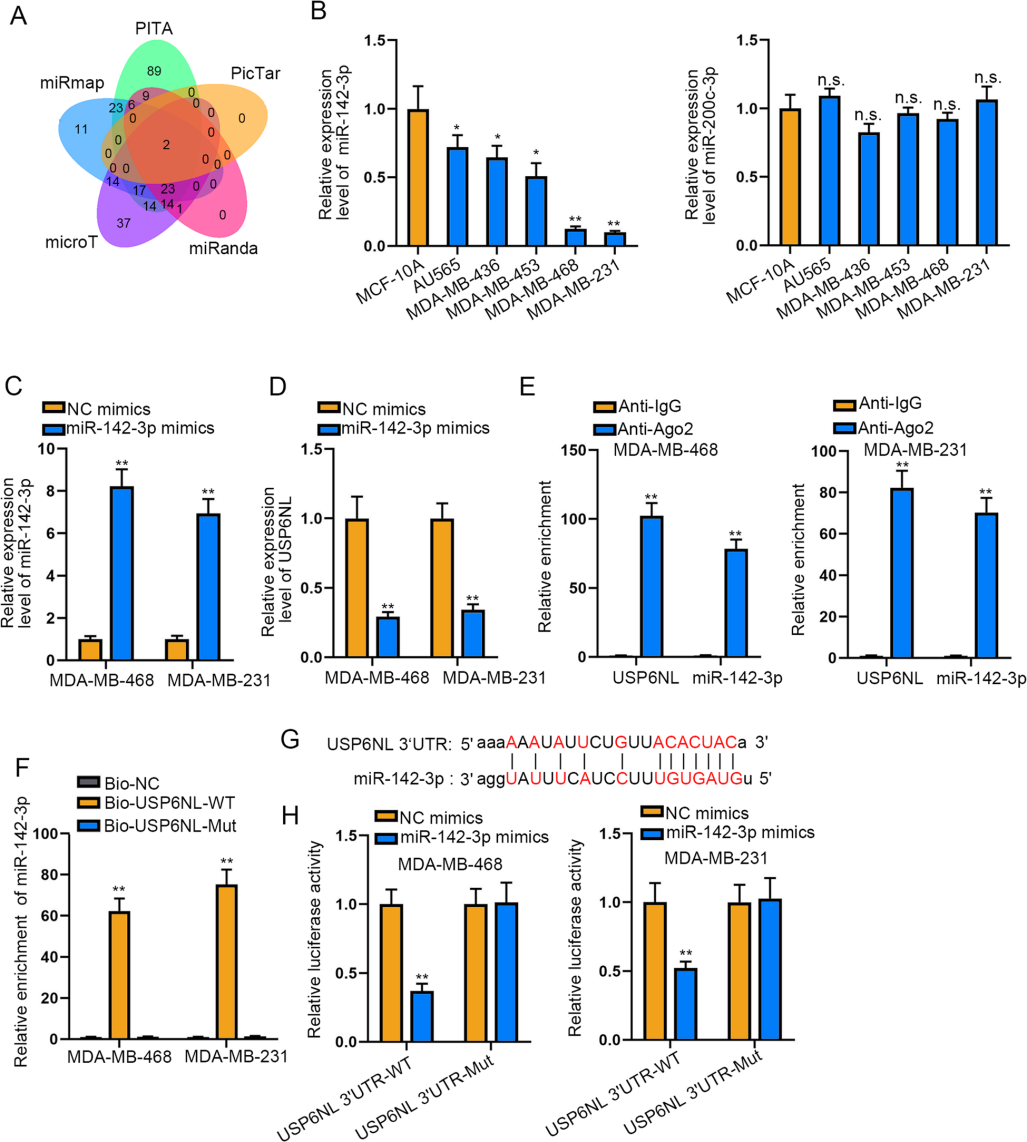
MiR-142-3p acts as the upstream of USP6NL in TNBC. **a**. Bioinformatics tools were used to predict candidate miRNAs binding to USP6NL. **b**. The expression of miR-142-3p/miR-200c-3p was examined by RT-qPCR in TNBC cells and normal MCF-10A cells. **c-d**. RT-qPCR detected miR-142-3p and USP6NL expression in TNBC cells transfected with miR-142-3p mimics. **e-f**. RIP assay and RNA pull down assay were carried out to verify the interplay between miR-142-3p and USP6NL. **g**. The binding sites of miR-142-3p to USP6NL’UTR were predicted by ENCORI. **h**. Luciferase reporter assay was applied to confirm the connection between miR-142-3p and USP6NL. *P < 0.05, **P < 0.01. n.s.: no significance

To prove the probable miRNAs upstream of USP6NL, five tools (including PITA, PicTar, miRanda, mircoT and miRmap) enclosed in ENCORI database (http://starbase.sysu.edu.cn/) were applied. Fortunately, two miRNAs shared by all above 5 tools were screened out as the potential miRNAs targeted UAP6NL (Fig. 2a). Further, we detected the expression level of both miRNAs in TNBC cells and discovered that miR-142-3p but not miR-200c-3p had low expression trend in TNBC cells compared with the normal MCF-10A cells (Fig. 2b). Thus, we elected miR-142-3p as the subject of follow-up experiments. Then we transfected miR-142-3p mimics into MDA-MB-468 and MDA-MB-231 cells and found an effective elevation on miR-142-3p expression in these two cells (Fig. 2c). Further, we uncovered that miR-142-3p up-regulation could decline the expression of both USP6NL mRNA and protein (Fig. 2d **and Supplementary Figure** **1****B**). Additionally, the results of RIP assay displayed that miR-142-3p and USP6NL were both abundant in anti-Ago2 group rather than Anti-IgG group (Fig. 2e), indicating their co-existence in Ago2-assmebled RNA-induced silencing complexes (RISCs). Meanwhile, the outcomes of RNA pull down assay unmasked the apparent enrichment of miR-142-3p by Bio-USP6NL-WT but not by bio-USP6NL-Mut (Fig. 2f), indicating the binding of miR-142-3p to USP6NL in TNBC cells. Under the analysis of ENCORI database, the binding sequence between miR-142-3p and USP6NL was exhibited in Fig. 2g. Moreover, miR-142-3p mimics sharply cut down the luciferase activity of USP6NL-WT, but not effected that of USP6NL-Mut (Fig. 2h). Taken together, miR-142-3p serves as the upstream of USP6NL in TNBC.

### MiR-142-3p plays an effective tumor-restraining part in TNBC

**Fig. 3:**
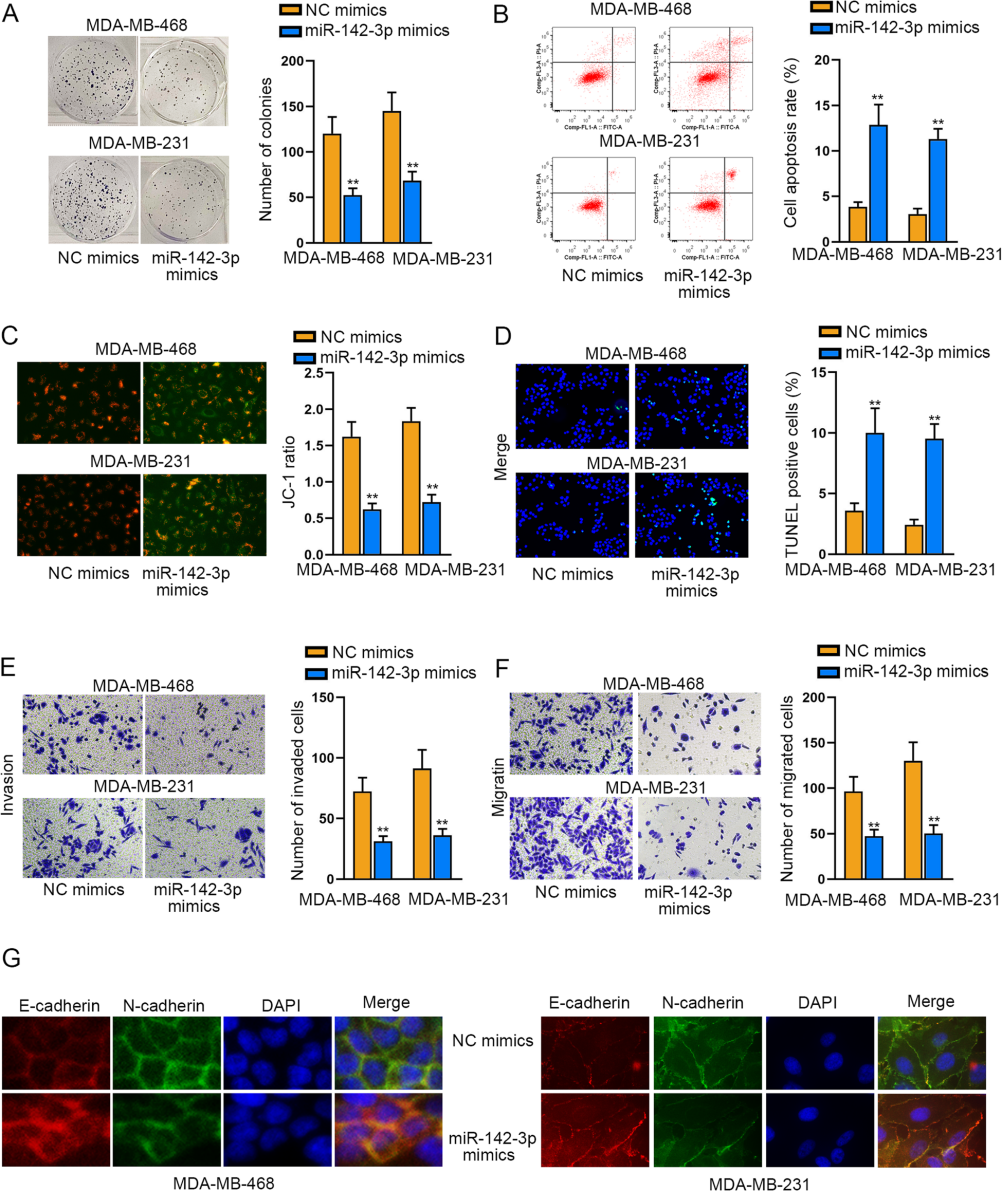
MiR-142-3p plays a tumor repressive part in TNBC. **a**. Colony formation assay was performed to assess the impact of miR-142-3p upregulation on cell proliferation. **b-d**. Under the context of miR-142-3p elevation, cell apoptosis was tested via flow cytometry analysis, JC-1 assay and TUNEL assay. The X axis of the FACS dot plots in Fig. 3b meant cells labelled with FITC and the Y axis indicated cells dyed by PI. **e-f**. The effect of enhanced miR-142-3p on cell migration and invasion was tested via transwell assays. **g**. IF assay analyzed the expression of two EMT-related proteins (E-cadherin and N-cadherin) in TNBC cells with or without miR-142-3p up-regulation. **P < 0.01

Afterwards, the effect of miR-142-3p on TNBC cellular behaviors was evaluated. As presented in Fig. 3a, the proliferation of TNBC cells was hampered in face of miR-142-3p elevation. In contrast, it manifested that ectopic expression of miR-142-3p accelerated cell apoptosis compared to NC mimics group (Fig. 3b-d). In the meantime, the capacities of cells to migrate and invade were overtly declined by miR-142-3p mimics (Fig. 3e-f). Similarly, overexpressed miR-142-3p also depressed the EMT progress in TNBC (Fig. 3g). Collectively, miR-142-3p is an effective cancer-suppressor in TNBC.

### LINC00689 is taken for a ceRNA to boost USP6NL by sponging miR-142-3p in TNBC

**Fig. 4:**
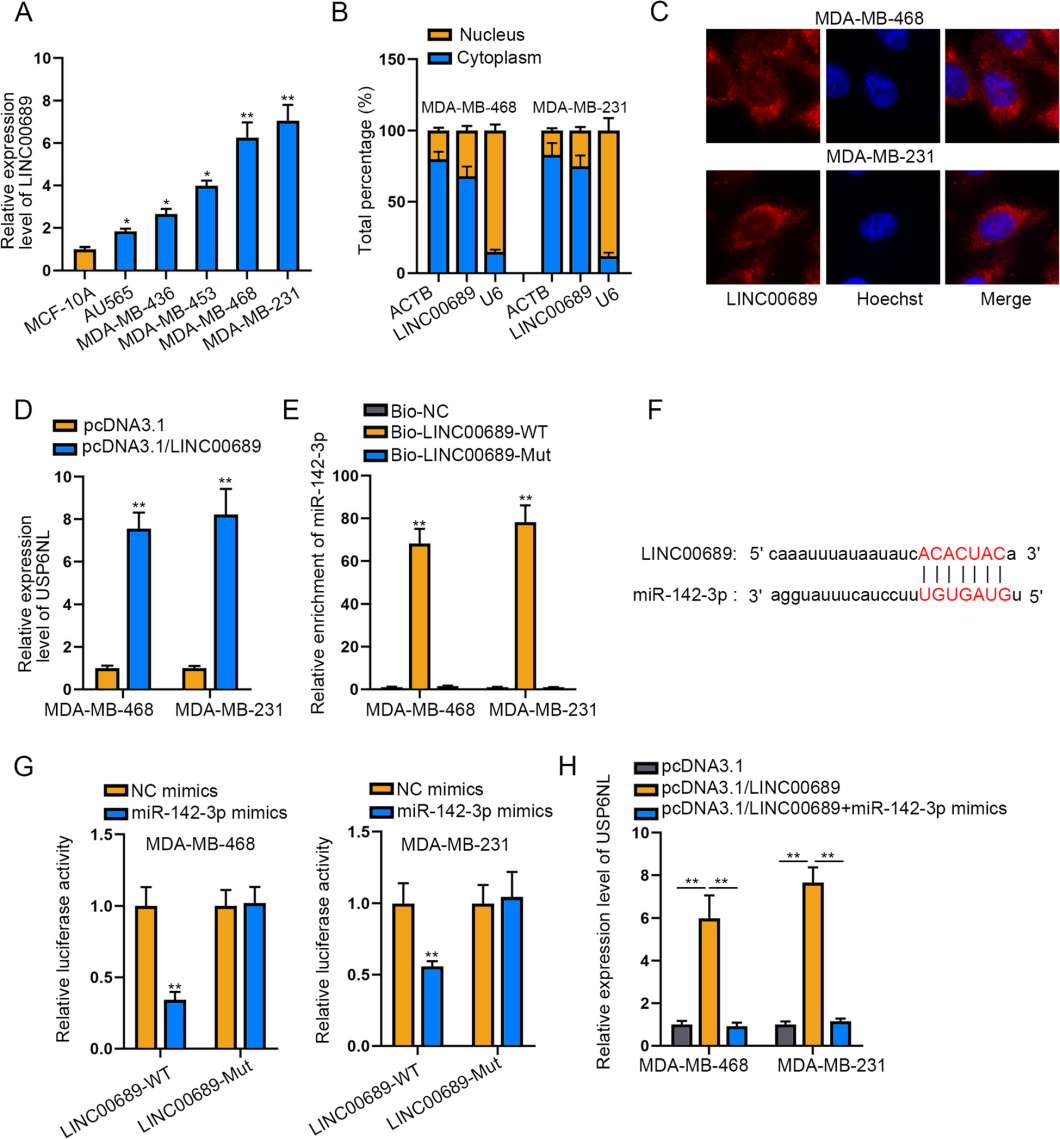
LINC00689 is taken for a ceRNA to regulate USP6NL by sponging miR-142-3p in TNBC. **a**. RT-qPCR detected the expression of LINC00689 in TNCB cells. **b-c**. The cytoplasmic location of LINC00689 in TNBC cells was identified by subcellular fraction assay and FISH assay. **d**. The expression of USP6NL in both cells was assessed by RT-qPCR in the case of LINC00689 overexpression. **e**. RNA pull down assay was used to confirm the interaction between LINC00689 and miR-142-3p. **f**. The binding sequence between LINC00689 and miR-142-3p was acquired via ENCORI. **g**. The combination between LINC00689 and miR-142-3p were identified by luciferase reporter assay. **h**. USP6NL expression was measured by RT-qPCR under the circumstance of overexpressed LINC00689 or together with miR-142-3p. *P < 0.05, **P < 0.01

Subsequently, we utilized ENCORI database (http://starbase.sysu.edu.cn/) to search for the potential lncRNAs interacting with miR-142-3p. As a result, total 61 lncRNAs were screened out, among which 22 lncRNAs were classed “8mer” that meant the sites matched well to the seed region of miR-142-3p. Then, we selected 6 lncRNAs (MATN1-AS1, ENTPD3-AS1, DUBR, LINC00689, PSMA3-AS1 and LINC00482) through excluding the unannotated ones as well as those have been studied in breast cancer. Further, the results of RNA pull down assay suggested that only LINC00689 among the selected 6 lncRNAs was highly concentrated in Bio-miR-142-3p group (**Supplementary Figure** **1****C**). Thereafter, we evaluated the expression of LINC00689 in TNBC cells. It was demonstrated that LINC00689 exhibited higher expression in TNBC cells than MCF-10A controls, with the highest level in MDA-MB-468 and MDA-MB-231 cells (Fig. 4a). As far as mechanism was concerned, lncRNAs are capable of affecting gene expression through post-transcriptional regulation via functioning as a competitive endogenous (ceRNA). According to findings of subcellular fractionation assay and FISH assay, LINC00689 was richly expressed in the cytoplasm of both TNBC cells (Fig. 4b-c). Next, both cells were transfected with pcRNA3.1/LINC00689. As a result, up-regulation of LINC00689 overtly promoted the levels of USP6NL mRNA and protein (Fig. 4d **and Supplementary Figure** **1****D**). Then the results of RNA pull down assay uncovered that USP6NL was effectively captured by Bio-LINC00689-WT instead of Bio-LINC00689-Mut in both TNBC cells (Fig. 4e). Additionally, the putative sites responsible for the interaction between LINC00689 and miR-142-3p were gained by ENCORI software (Fig. 4f). Furthermore, it was displayed that elevating miR-142-3p obviously cut down the luciferase activity of LINC00689-WT but not that of LINC00689-Mut (Fig. 4g). In the last step, rescue assays helped us discover the influence of LINC00689/miR-142-3p pathway on USP6NL. As anticipated, the mRNA and protein levels of USP6NL were both up-regulated by LINC00689 overexpression, but then cut down by miR-142-3p elevation (Fig. 4h **and Supplementary Figure** **1****E**). Overall, LINC00689 is taken for a ceRNA to upregulate USP6NL by sponging miR-142-3p in TNBC.

### LINC00689 affects TNBC cell proliferation and apoptosis through miR-142-3p/USP6NL axis

**Fig. 5:**
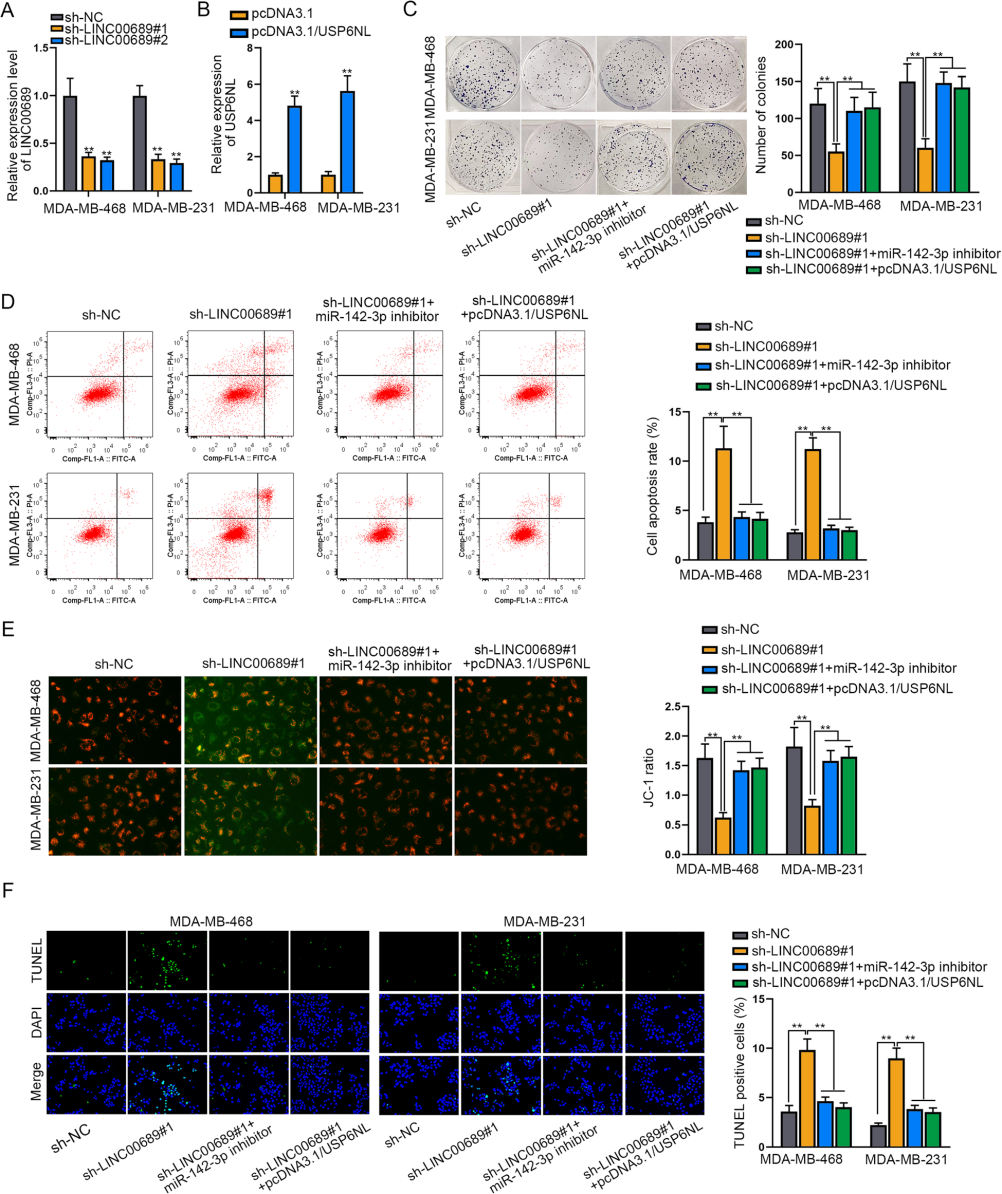
MiR-142-3p suppression or USP6NL overexpression abolishes the effect of LINC00689 silence on TNBC cell proliferation and apoptosis. **a**. The expression of LINC00689 was measured by RT-qPCR in the context of LINC00689 knockdown. **b**. RT-qPCR determined the expression of USP6NL in TNBC cells transfected with pcDNA3.1 or pcDNA3.1/USP6NL. **c**. The colony formation assay detected the effect of inhibited miR-142-3p or upregulated USP6NL on LINC00689 silence-mediated cell proliferation. **d-f**. Flow cytometry analysis, JC-1 assay and TUNEL assay evaluated the apoptosis of cells under diverse conditions. The X axis of the FACS dot plots in Fig. 5d meant cells labelled with FITC and the Y axis indicated cells dyed by PI. **P < 0.01

Thereafter, we conducted rescue assays to illustrate the regulatory function of LINC00689/miR-142-3p/USP6NL pathway in TNBC. Firstly, we observed that the expression of LINC00689 was apparently silenced by sh-LINC00689#1/#2 in both MDA-MB-468 and MDA-MB-231 cells (Fig. 5a)**.** Also, transfection of miR-142-3p inhibitor effectively decreased the expression of miR-142-3p in these two TNBC cells (**Supplementary Figure** **1****F**). In the meantime, the expression of USP6NL was dramatically elevated by transfecting with pcDNA3.1/USP6NL (Fig. 5b **and Supplementary Figure** **1****G**). Next, the results of colony formation assay Indicated that the inhibition on TNBC cell proliferation resulted from LINC00689 knockdown was fully rescued by miR-142-3p suppression or USP6NL up-regulation (Fig. 5c). Meanwhile, silencing LINC00689 led to stimulation on TNBC cell apoptosis, while inhibited miR-142-3p or over-expressed USP6NL absolutely reversed such impact (Fig. 5d-f). Overall, miR-142-3p and USP6NL were involved in LINC00689-regulatedTNBC cell proliferation and apoptosis.

### LINC00689 contributes to TNBC cell migration, invasion and EMT via miR-142-3p/USP6NL signaling

**Fig. 6:**
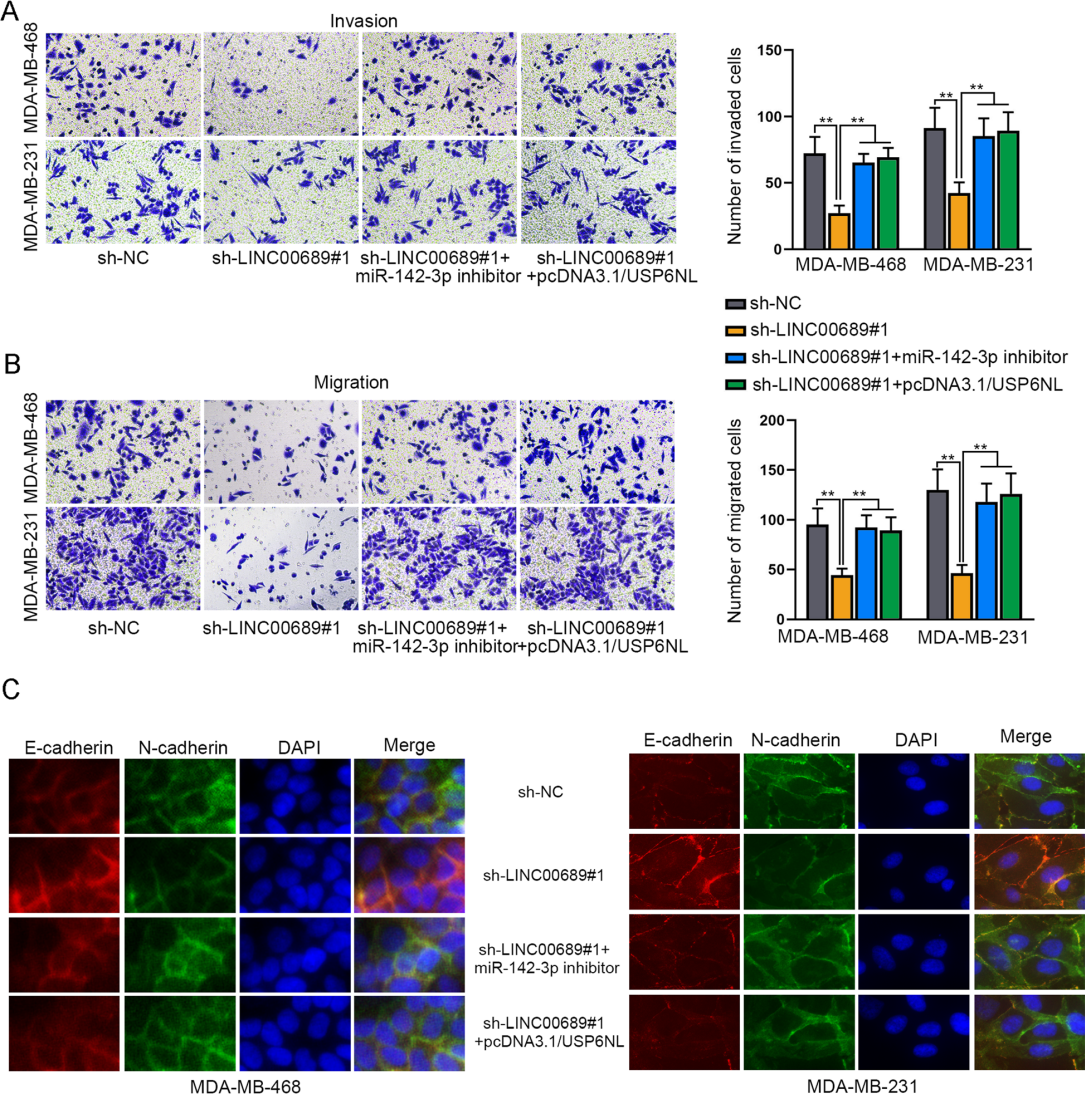
MiR-142-3p suppression or USP6NL overexpression abolishes the effect of LINC00689 silence on TNBC cell migration, invasion and EMT. **a-b**. Transwell assay was implemented to test cell migration and invasion in response to different contexts. **c**. IF assay assessed the EMT progress in indicated TNBC cells. **P < 0.01

Next, we continued to detect the influence of this pathway on TNBC cell migration, invasion and EMT. Through conducting transwell assays, we unveiled that cell motility was restrained owing to LINC00689 down-regulation, while such restraining effect was totally restored owing to miR-142-3p suppression or USP6NL overexpression (Fig. 6a-b). Following, in the outcomes of IF assays, LINC00689 depletion retarded EMT progression was reversed on account of miR-142-3p inhibition or USP6NL enhancement (Fig. 6c). In a word, LINC00689 depends on miR-142-3p/USP6NL signaling to work as a facilitator in TNBC.

## Discussion

TNBC, one subtype of breast cancer, has been paid increasing attention due to high occurrence and mortality. Prevalently, targeted therapy has become a hot topic in diverse cancers, including TNBC. In this regard, promising molecules suitable for such therapy have caught researchers’ eyes in recent years, especially protein-coding genes. For instance, MCP-1 was suggested to play a key role in TNBC by affecting tumor invasiveness and metastasis [17]. Strong expression of CDCA7 was indicated to have correlations with metastatic relapse status and shorted the survival time of TNBC patients [18]. Similarly, RAB1A knockdown suppressed cell proliferation and EMT in TNBC [19]. Presently, we unveiled that the expression of USP6NL was enhanced in TNBC cells, and the elevated expression of USP6NL contributed to cell proliferation, migration and invasion as well as EMT in TNBC cells. Thus, we deduced that USP6NL served a cancer-facilitating part in TNBC, suggesting it as an effective and useful biomarker for TNBC treatment.

Since the mRNAs of protein-coding genes could be recognized and silenced by miRNAs, we then made use of bioinformatics tools to find out probable miRNAs upstream of USP6NL. Herein, miR-142-3p was finally proved to target USP6NL in TNBC. Moreover, we disclosed that miR-142-3p up-regulation had similar effects like silencing USP6NL on TNBC cellular behaviors. In other word, miR-142-3p worked as a cancer-repressor in TNBC. Previously, miR-142-3p has also been indicated to participate in cancer development by a similar manner. For instance, in colorectal cancer, miR-142-3p inhibited CDK4 to restrain cell growth [20]. Besides, miR-142-3p also regulated the progression of cervical cancer and gastric cancer [21, 22].

Nowadays, more and more evidence disclosed that long non-coding RNAs (lncRNAs) are also implicated in TNBC development. For example, lncRNA BORG accelerated the survival and chemoresistance of TNBC cells [23]. LncRNA PVT1 mediated TNBC progression via KLF5/beta-catenin signaling [24]. Generally speaking, lncRNAs are able to be a ceRNA to regulate mRNA expression by competitively combining with miRNAs [25, 26]. As for LINC00689, Liu et al. explained that LINC00689 might affect glioma progression by regulating miR-338-3p/PKM2 pathways [27]. In current study, LINC00689 was recognized as the upstream of miR-142-3p and worked as a ceRNA to boost USP6NL expression by sequestering miR-142-3p. In the end, rescue assays proofed that inhibited miR-142-3p or up-regulated USP6NL could absolutely reverse the suppression of depleted LINC00689 on TNBC cell proliferation, migration/invasion and EMT.

## Conclusion

Our results showed that USP6NL mediated by LINC00689/miR-142-3p promotes the development of TNBC. These findings indicated that USP6NL might be a novel and promising therapeutic biomarker for TNBC.

## Supplementary information

**Additional file 1: Supplementary Figure S1** LINC00689 elevates USP6NL expression in TNBC cells via miR-142-3p. A-B. Western blot tested the protein level of USP6NL in MDA-MB-468 and MDA-MB-231 cells under USP6NL inhibition or miR-142-3p upregulation. C. RNA pull down assay analyzed the interaction of miR-142-3p with indicated 6 lncRNAs in MDA-MB-468 cells. D-E. USP6NL protein expression was examined by western blot in two TNBC cells with LINC00689 overexpression or together with miR-142-3p inhibition. F. RT-qPCR determined the inhibition efficiency of miR-142-3p in these two cells. G. Western blot proved the indeed upregulation of USP6NL protein in cells transfected with pcDNA3.1/USP6NL. **P < 0.01.

**Additional file 2.** The original, unprocessed versions of all western blots.

## Data Availability

The datasets used and/or analysed during the current study available from the corresponding author on reasonable request.

## Abbreviations

TNBC: Triple-negative breast cancer
lncRNAs: Long non-coding RNAs
mRNA: Messenger RNA
ceRNAs: Competing endogenous RNAs
miRNA: MicroRNA
USP6NL: Ubiquitin specific peptidase 6 N-terminal like
LINC00689: Long intergenic non-protein coding RNA 689
EMT: Epithelial-mesenchymal transitions
3’UTR: 3′-untranslated region
ATCC: American Type Culture Collection
DMEM: Dulbecco’s modification of Eagle’s medium
FBS: Fatal Bovine Serum
TUNEL: Terminal deoxynucleotidyl transferase (TdT) dUTP Nick-End Labeling
DAPI): 4′,6-diamidino-2-phenylindole
Wt: Wild-type
Mut: Mutant
IF: Immunofluorescence
FISH: Fluorescence in situ hybridization
CRC: Colorectal cancer
RT-qPCR: Real-time quantitative PCR
RIP: RNA immunoprecipitation
SD: Standard deviation
ANOVA: Analysis of Variance
