# Supplementary figures and images for: USP6NL mediated by LINC00689/miR-142-3p promotes the development of triple-negative breast cancer
Source: BMC Cancer. 2020 Oct 14;20:998. doi: 10.1186/s12885-020-07394-z (PMC7559130; doi:10.1186/s12885-020-07394-z)

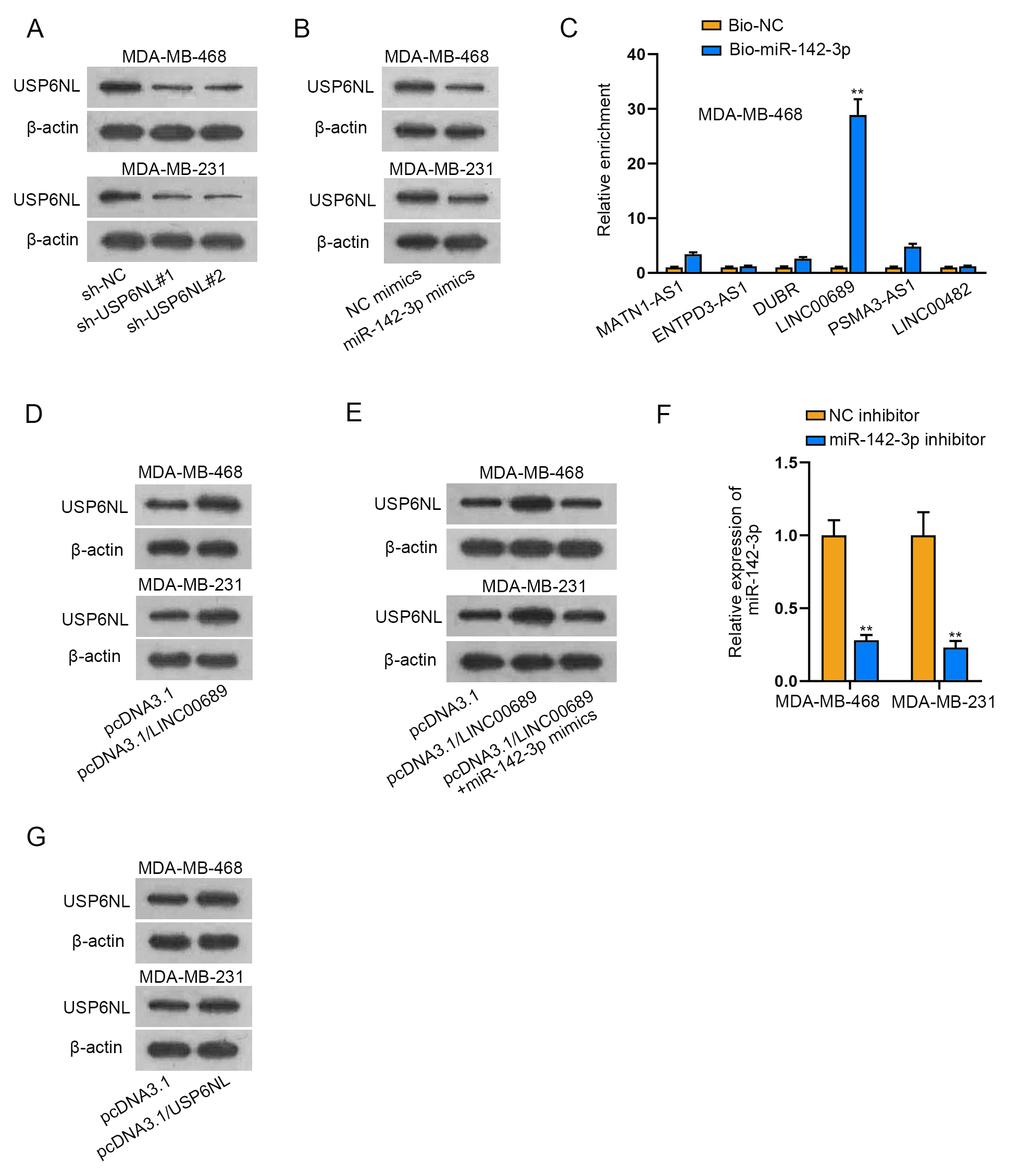

Supplement: Supplementary file 1 — Additional file 1: Supplementary Figure S1 LINC00689 elevates USP6NL expression in TNBC cells via miR-142-3p. A-B. Western blot tested the protein level of USP6NL in MDA-MB-468 and MDA-MB-231 cells under USP6NL inhibition or miR-142-3p upregulation. C. RNA pull down assay analyzed the interaction of miR-142-3p with indicated 6 lncRNAs in MDA-MB-468 cells. D-E. USP6NL protein expression was examined by western blot in two TNBC cells with LINC00689 overexpression or together with miR-142-3p inhibition. F. RT-qPCR determined the inhibition efficiency of miR-142-3p in these two cells. G. Western blot proved the indeed upregulation of USP6NL protein in cells transfected with pcDNA3.1/USP6NL. **P < 0.01. [file 12885_2020_7394_MOESM1_ESM.tif]

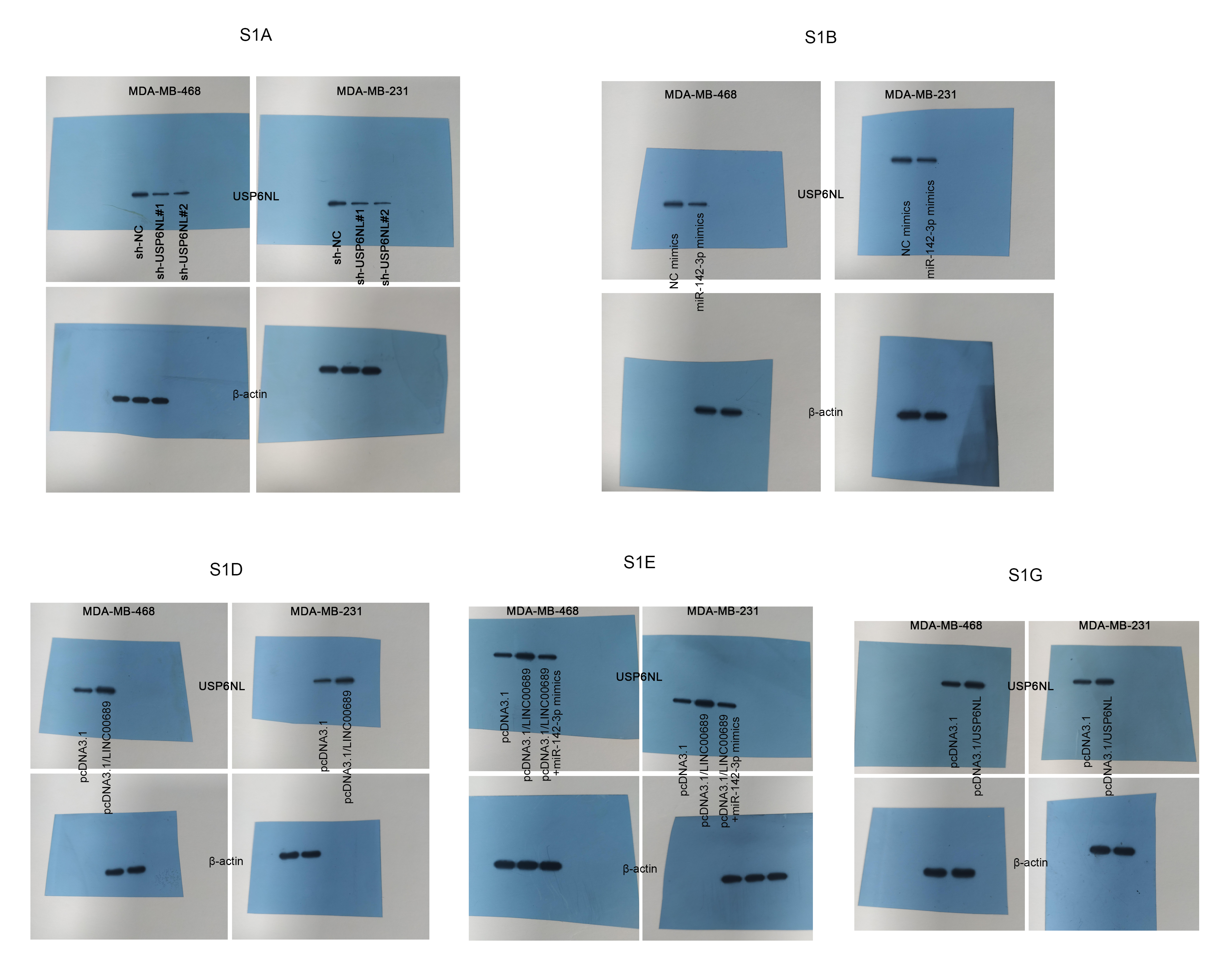

Supplement: Supplementary file 2 — Additional file 2. The original, unprocessed versions of all western blots. [file 12885_2020_7394_MOESM2_ESM.tif]
